# Monosomes buffer translational stress to allow for active ribosome elongation

**DOI:** 10.3389/fmolb.2023.1158043

**Published:** 2023-05-26

**Authors:** Rico Schieweck, Giuliana Ciccopiedi, Kenneth Klau, Bastian Popper

**Affiliations:** ^1^ Biomedical Center (BMC), Department for Cell Biology and Anatomy, Medical Faculty, Ludwig-Maximilians-University, Munich, Germany; ^2^ Biomedical Center (BMC), Core Facility Animal Models, Ludwig-Maximilians-University, Munich, Germany

**Keywords:** translation elongation, starvation, human cells, translation speed, polysome profiling, eEF2, cellular stress, mTOR

## Abstract

**Introduction:** The synthesis of proteins is a fundamental process in the life-span of all cells. The activation of ribosomes on transcripts is the starting signal for elongation and, in turn, the translation of an mRNA. Thereby, most mRNAs circulate between single (monosomes) and multi ribosomal particles (polysomes), a process that defines their translational activity. The interplay between monosomes and polysomes is thought to crucially impact translation rate. How monosomes and polysomes are balanced during stress remains, however, elusive.

**Methods:** Here, we set out to investigate the monosome and polysome levels as well as their kinetics under different translational stress conditions including mTOR inhibition, downregulation of the eukaryotic elongation factor 2 (eEF2) and amino acid depletion.

**Results:** By using a timed ribosome runoff approach in combination with polysome profiling, we found that the used translational stressors show very distinct effects on translation. However, they all had in common that the activity of monosomes was preferentially affected. This adaptation seems to be needed for sufficient translation elongation. Even under harsh conditions such as amino acid starvation, we detected active polysomes while monosomes were mostly inactive. Hence, it is plausible that cells compensate the reduced availability of essential factors during stress by adapting the levels of active monosomes to favor sufficient elongation.

**Discussion:** These results suggest that monosome and polysome levels are balanced under stress conditions. Together, our data argue for the existence of translational plasticity that ensure sufficient protein synthesis under stress conditions, a process that is necessary for cell survival and recovery.

## 1 Introduction

Protein synthesis is a highly regulated process that is essential to maintain protein homeostasis (proteostasis) (Schieweck et al., 2016; Hanson and Coller, 2018). Therefore, cells have evolved numerous regulators such as RNA-binding proteins (Schieweck et al., 2021a), kinases (Leprivier et al., 2013) and chaperones (Popper et al., 2021) to control translation. Research in the last years has shown that the ribosome itself can regulate translation of certain transcripts (Xue and Barna, 2012). Thereby, ribosomal proteins can recruit ribosomes to certain transcripts (Kondrashov et al., 2011). However, translation is also regulated by the levels of ribosomes influencing a subset of mRNAs (Khajuria et al., 2018). This finding suggests that transcripts require different numbers of ribosomes for efficient translation. Supportive for this notion is the finding by Cheng et al. (2019) showing that the deficiency of large and small ribosomal subunits has distinct impacts on gene expression. Together, these findings suggest that ribosomal levels can actively regulate translation.

In addition to their levels, ribosomes also regulate protein expression by their velocity (Kapur et al., 2017). Here, the speed of ribosomes critically determines co-translational folding trajectories of some proteins in order to allow them to find their native structure (Kirchner et al., 2017; Lakshminarayan et al., 2020; Rauscher et al., 2021) and to assemble into protein complexes (Shiber et al., 2018). Pathological mutations that alter ribosome speed contribute to severe disorders such as neurodegeneration (Lee et al., 2006; Ishimura et al., 2014) or cystic fibrosis (Kirchner et al., 2017). These findings clearly show that some mRNAs require a certain translational activity and ribosome speed to produce sufficient levels of functional proteins. This finding is particularly important for translational stress that causes global changes in translational activity (Klann et al., 2020). How ribosomes respond to translational stress has been mainly investigated at the level of translational activity.

Here, we set out to systematically investigate how ribosomes respond to translational stress conditions. Therefore, we exploited distinctly timed Harringtonine pulses to freeze initiating ribosomes and to monitor the kinetics of monosomes and polysomes. To address how the translatome reacts to stress, we exploited three different stress paradigms: inhibition of mTOR, downregulation of eEF2 and amino acid depletion. We found that even though all three stressors had different impacts on the translatome, they all preferentially affect monosomes and reduce their activity. In contrast, polysome activity was only impacted under harsh stress condition such as amino acid depletion. These data suggest that cells buffer the reduced availability of essential translational factors by favoring polysomes. This, in turn, allows for the selection of transcript for translation as part of cellular survival pathways. Together, our data provide a ribosome-centered explanation for ongoing translation under stress conditions.

## 2 Materials and methods

### 2.1 Cell culture

HEK293T cells were cultured in DMEM medium supplemented with 10% FCS. For polysome profiling or western blot analysis, 1.5 million cells were seeded one day before experimentation. For eEF2 downregulation experiments, 750.000 cells were seeded, transfected with either shControl or sheEF2 constructs and incubated for 2 days.

### 2.2 shRNA construct generation and transfection

The following sequences were used to downregulate eEF2:

shEEF2_1: 5′- gat​ccc​cGC​CAT​CCG​CCA​CCA​TGG​TGt​tca​aga​gaC​ACC​ATG​GTG​GCG​GAT​GGC​ttt​ttc -3′

shEEF2_2: 5′- gat​ccc​cCG​GGG​TGT​GCG​CTT​CGA​CGt​tca​aga​gaC​GTC​GAA​GCG​CAC​ACC​CCG​ttt​ttc -3′

They were cloned in the pSuperior expression vector system that expressed GFP to control transfection using XhoI and BglII. 9 μg of plasmid was used to transfect cells exploiting the calcium phosphate method (Goetze et al., 2004).

### 2.3 Western blot analysis

For immunoblotting, cells were washed once with warm PBS and lysed in hot SDS loading buffer. For protein isolation from sucrose gradients, proteins were isolated using methanol-chloroform extraction (Wessel and Flügge, 1984) and dissolved in SDS buffer overnight at room temperature. Proteins were separated by SDS-PAGE and transferred to a nitrocellulose membrane (pore size 0.2 µm). Membranes were first blocked in blocking solution (2% (w/v) BSA, 0.1 vol% Tween 20, 0.1% (w/v) sodium azide in 1 × TBS pH 7.5) for at least 1 h or overnight. Proteins were detected using the following antibodies: anti-eEF2, anti-p-eEF2, anti-mTOR, anti-p-mTOR, [1:1.000 dilution, all from rabbit, Cell Signaling, Germany]; rabbit anti-Rps6 [1:1.000 dilution, Sigma, Germany], rabbit anti-p-Rps6 [1:1.000 dilution, Cell Signaling, Germany]; rabbit anti-Rpl7a [1:1.000 dilution, Abcam, Germany]; rabbit anti-α-Tubulin [1:5.000 dilution, Abcam, Germany], mouse anti-ACTB [1:5.000 dilution, Sigma, Germany] and anti-puromycin [1:10.000 dilution, clone 12D10 Millipore, Germany]. The antibodies were diluted in blocking solution and incubated with the membrane overnight. Primary antibody binding was detected using IRdye labeled secondary anti-mouse or anti-rabbit antibodies (1:10.000 dilution, Licor) and fluorescence signal was detected with an Odysee scanner (Licor, Germany). For western blot analysis, band intensities were quantified with Image Studio Lite (version 5). Band signals were normalized to the loading control to allow for comparison between different conditions.

### 2.4 Polysome profiling

Polysome profiling was performed as previously published (Schieweck et al., 2021c). In brief, cells were scrapped in polysome lysis buffer [150 mM NaCl, 5 mM MgCl_2_, 10 mM Tris-HCl pH 7.4, 1 vol% NP-40, 1% (w/v) sodium deoxycholate supplemented with 100 μg/mL cycloheximide (CHX), 2 mM dithiothreitol, DTT and RNase inhibitor]. Lysates were loaded on a sucrose gradient [18%–50% (w/v) sucrose] and spun at 35.000 rpm (SW55Ti rotor, Beckman) for 1.5 h at 4°C. Gradients were then fractionated using a piston gradient fractionator (Biocomp, Germany). RNA fate was detected using an UV lamp at 254 nm.

### 2.5 Translation kinetics

For translation kinetics, cells were incubated with 2 μg/mL HRN (Biomol, Germany) for 1, 5, and 10 min. Ribosome elongation was stopped by adding 100 μg/mL CHX (Roth, Germany). For the time point *t* = 0, cells were incubated with CHX (Roth, Germany) for 10 min. CHX and HRN were dissolved in DMSO. Cells were then washed in warm PBS supplemented with 100 μg/mL CHX, lysed in polysome lysis buffer and proceeded for polysome profiling.

### 2.6 Puromycylation

Puromycylation was performed as previously described (Gao et al., 2015; Schieweck et al., 2021b). In brief, HEK cells were incubated with 2 μg/mL HRN for 1, 5, and 10 min as described above. Nascent chains were subsequently labeled with 25 µM PMY for 10 min. As control (time point “0”), cells were incubated with 25 µM PMY for 10 min. Upon puromycylation, HEK cells were washed in warm PBS and lysed in hot SDS lysis buffer and subjected to western blot analysis. To analyze puromycylation experiments, PMY signal for the different time points was normalized to α Tubulin and fold changes calculated to time point “0”. Fold changes were then plotted against the duration of HRN treatment and fitted using an exponential decay kinetics: *Y* = (Y0-Plateau)·e (-k·x) + Plateau. Prism software (version 5 GraphPad, San Diego, CA, United States) was used for analysis.

### 2.7 Analysis of translation kinetics

**FIGURE 1:**
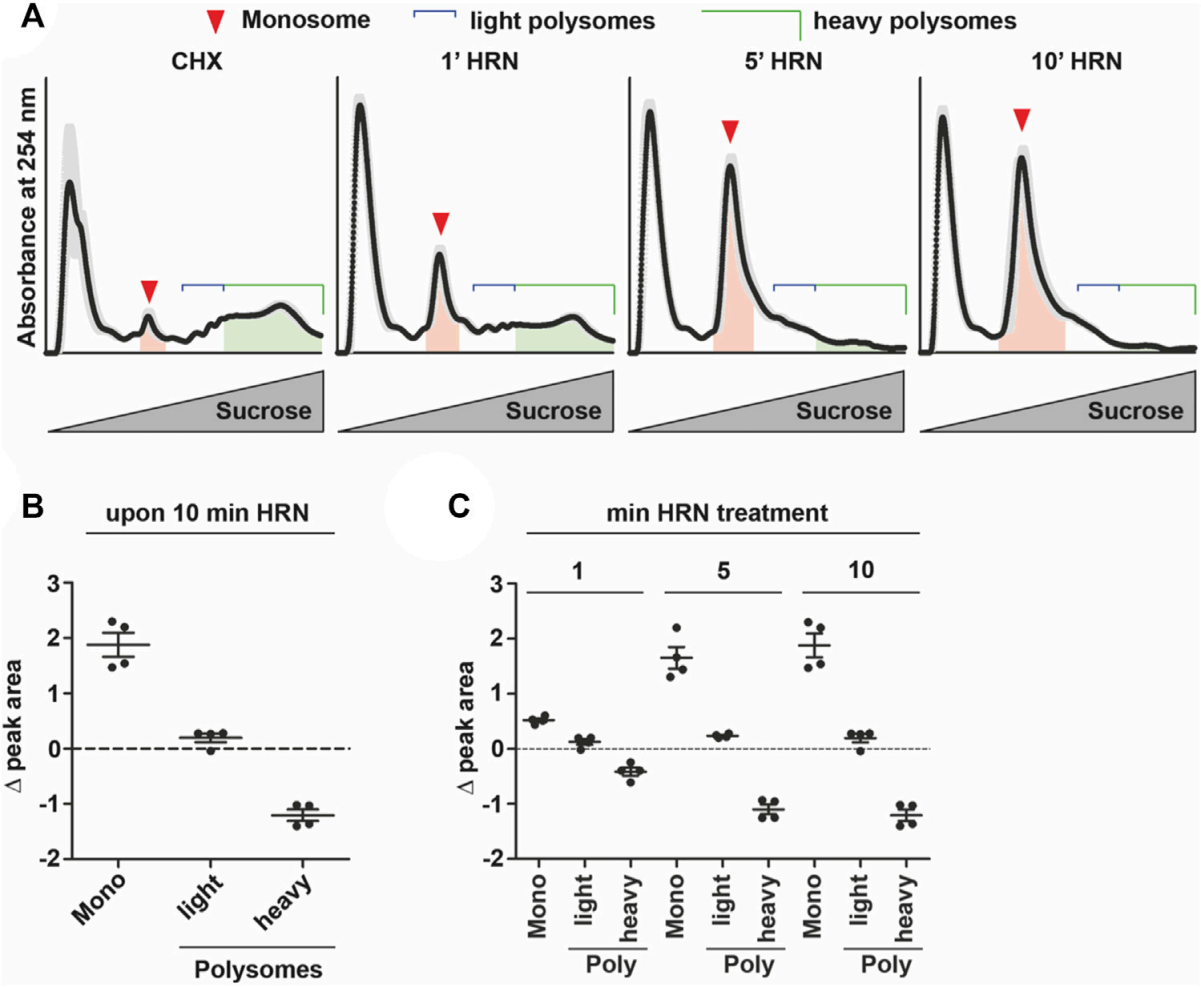
Translation kinetics of HEK293T cells. **(A)** Overlay of polysome profiles from four independent experiments during the time course of ribosome run-off. Shadows represent SEM. Areas under the curve for monosomes and heavy polysomes, respectively, are indicated. **(B,C)** Changes in peak areas of monosomes, light and heavy polysomes upon 10 min of HRN incubation **(B)** and for all time points investigated **(C)**. Dots represent biological replicates, *n* = 4, data is represented as mean ± SEM.

Monosome peaks were identified based on their migration characteristics in polysome profiles as well as according to their increase during ribosome runoff. A constant distance of 8.9 mm between monosome and polysome peak was applied to identify heavy polysomes. Ribosomes that accumulate between monosome and heavy polysomes were considered as light polysomes. To analyze translation kinetics, areas under the curve of the monosome and heavy polysome peaks were calculated based on their absorbance profile at 254 nm (Figure 1A). For polysome profiling, baseline was set using 18% (w/v) sucrose solution. Importantly, RNA signal in polysome fractions upon runoff was more than 25 times higher than the absorbance signal of 18% (w/v) sucrose. For kinetic experiments, the ratio of polysome to monosome peak area was calculated. For monosome and polysome kinetics, fold changes of monosome and polysome peak areas relative to *t* = 0 were calculated, respectively. To determine rate constants, P/M ratios as well as monosome and polysome fold changes were plotted against the duration of HRN treatment. The curves for translation and polysome kinetics were fitted using a one phase decay kinetics: *Y* = (Y0-Plateau)·e (-k·x) + Plateau. For monosome kinetics a plateau followed by one phase association was used: *Y* = IF{X < X0, Y0, Y0 + (Plateau-Y0)·[1-e (-k·x-x0)]}. For ribosome net flux kinetics, we computed the differences between Monosome (ΔM) and Polysome (ΔP) areas for the different HRN time points to the CHX control profile, respectively. To calculate the ribosome net flux, we calculated the sum of ΔM and ΔP and plotted these values over the HRN incubation time. For analysis, prism software (version 5 GraphPad, San Diego, CA, United States) was used.

### 2.8 Statistics

To calculate *p*-values and fit data, the prism software (version 5 GraphPad, San Diego, CA, United States) was used. For statistical analysis, Student’s *t*-test, one-sample *t*-test or One-way ANOVA followed by Tukey’s Multiple Comparison Test were used. *p* < 0.05 was considered as statistically significant if not stated otherwise.

## 3 Results

**FIGURE 2:**
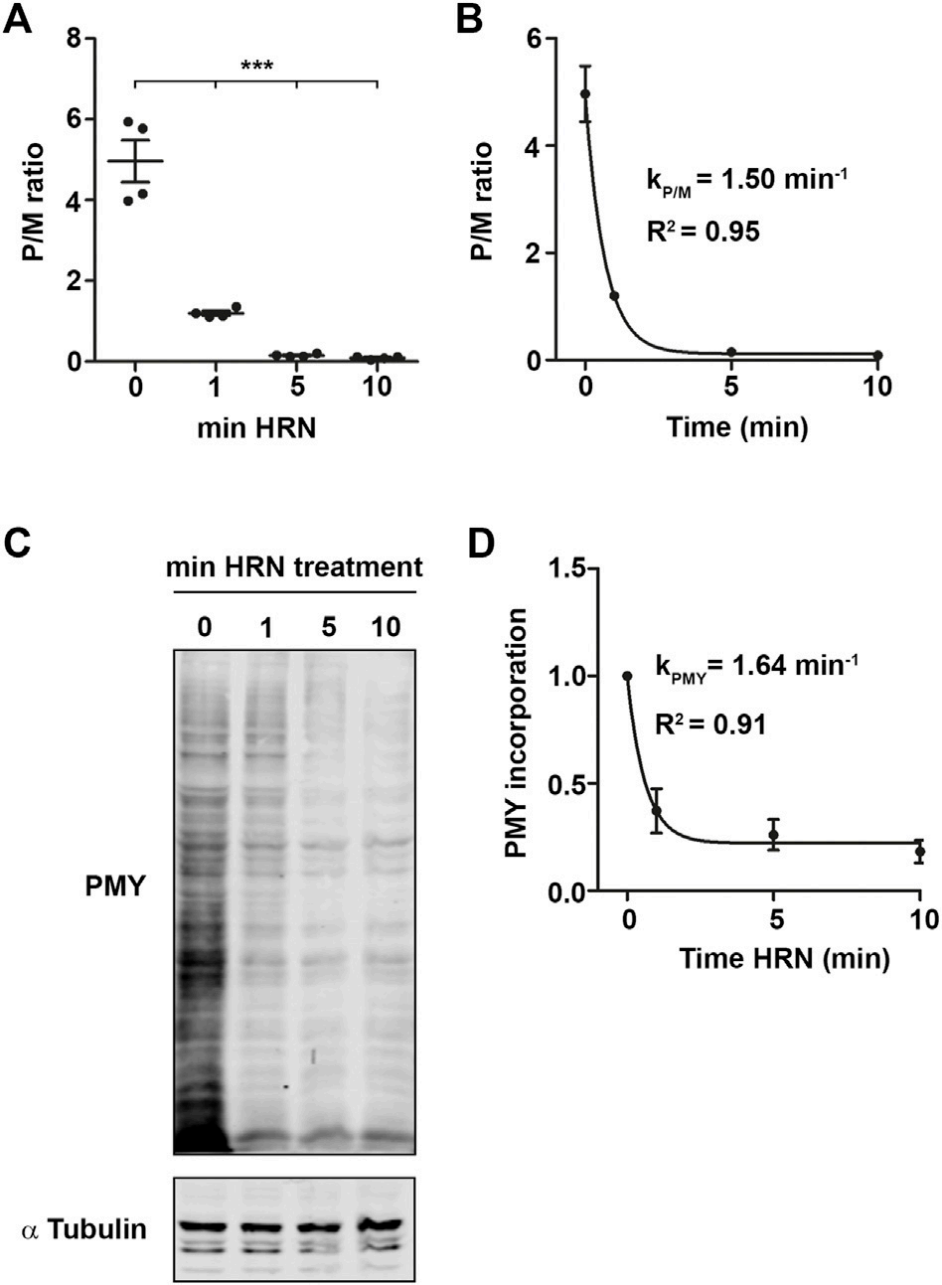
Nascent chain kinetics confirm translation kinetics experiments. **(A)** Polysome-to-monosome (P/M) ratios for the different HRN incubation time points. **(B)** P/M ratios as in **(A)** fitted using an exponential decay kinetics. **(C,D)** Representative immunoblot against PMY for different time points **(C)** and quantification **(D)**. α Tubulin was used as loading control. Dots represent independent biological replicates. *p*-values were calculated using One-way ANOVA with Tukey’s Multiple Comparison test. ***p < 0.001, *n* = 3 for puromycylation, *n* = 4 for translation kinetics, data is represented as mean ± SEM.

To address translation kinetics of cells, we used a combination of the translation inhibitors cycloheximide (CHX) and Harringtonine (HRN). CHX is known to stall elongating ribosomes (Schneider-Poetsch et al., 2010). HRN freezes only initiating ribosomes at the start codon (Ingolia et al., 2011) but does not affect translation elongation or early steps of initiation. Thereby, it allows to distinguish between monosome and polysome levels as well as speed (Ingolia et al., 2011). We incubated HEK293T cells with HRN and stopped the elongation after 1, 5, and 10 min by adding CHX. We chose these time points as they allowed us to investigate the complete runoff of the cellular translatome. Then, we performed polysome profiling to detected monosome and polysome levels. Polysome profiling is a well-established technique to isolate ribosomal complexes (Supplementary Figures S1A, B). As expected, we observed that extending the incubation time with HRN resulted in a depletion of polysomes and an increase in the monosome peak (Figure 1A). As a next step, we investigated the extent of how much monosomes and polysomes are changing comparing CHX and 10 min HRN treated cells by computing the differences in peak areas. Therefore, we focused on monosome, light and heavy polysomes. We defined “light polysomes” as mRNAs containing 2–4 ribosomes and “heavy polysomes” as transcripts containing more than 4 ribosomes (Figure 1A). We observed that monosomes and heavy polysomes are changing (Figures 1B, C). Interestingly, the peak area of light polysomes showed less pronounced changes during ribosome runoff (Figures 1B, C) even though the profiles showed clear altered shapes in this region (Figure 1A). This might be due to the accumulation of slowly translating mRNAs. Another, not mutually exclusive possibility is the occurrence of stalled/colliding ribosomes that might accumulate in light polysomes (Wu et al., 2020). Next, we wanted to analyze the dynamics of ribosome kinetics. Therefore, we calculated the changes in monosome, light and heavy polysome areas during HRN incubation (Figure 1C). We found that the monosome peak instantly increases with decreasing heavy polysome levels (Figure 1C). Based on this finding, we concluded that ribosome termination and recycling are not rate limiting steps during ribosome runoff in our assay. Importantly, we found that the majority (65% ± 5%) of the increase in monosomes (upon 10 min of HRN) is mediated by the initiation of ribosomes that underwent elongation in heavy polysomes. We speculated that initiation of yet translationally silent mRNAs would account for the additional 35%. Another, not mutually exclusive possibility is the formation of dormant ribosomes under these conditions (Shetty et al., 2023). Importantly, we observed that the vast majority of ribosomes (4 times more) are found in polysomes (Figure 2A). Together, these observations strongly suggest that the majority of ribosomes that underwent active translation, recycle and reinitiate translation during our runoff experiments. To approximate the speed of translation, we calculated the polysome-to-monosome (P/M) ratio during HRN incubation (Figure 2A). As a next step, we fitted these values with an exponential decay kinetics model (Argu et al., 2018) (Figure 2B). Importantly, our kinetics data fit well with the exponential decay kinetics as indicated by the high coefficient of determination value *R* ^2^ (Figure 2B). To complement our method, we performed puromycylation. Puromycylation is the method of choice to investigate translation at the level of nascent proteins (Schmidt et al., 2009). To this end, we incubated HEK cells with HRN to induce ribosome runoff and labeled nascent chains after 1, 5, and 10 min for 10 min with puromycin (PMY). As expected, we observed a decrease in PMY-labeled proteins during ribosome runoff (Figures 2C, D). Moreover, we calculated the PMY fold change during ribosome runoff and fitted these values with the same exponential decay kinetics. We observed similar *R* ^2^ value and kinetic constant supporting our polysome profiling approach (compare Figures 2B, D).

**FIGURE 3:**
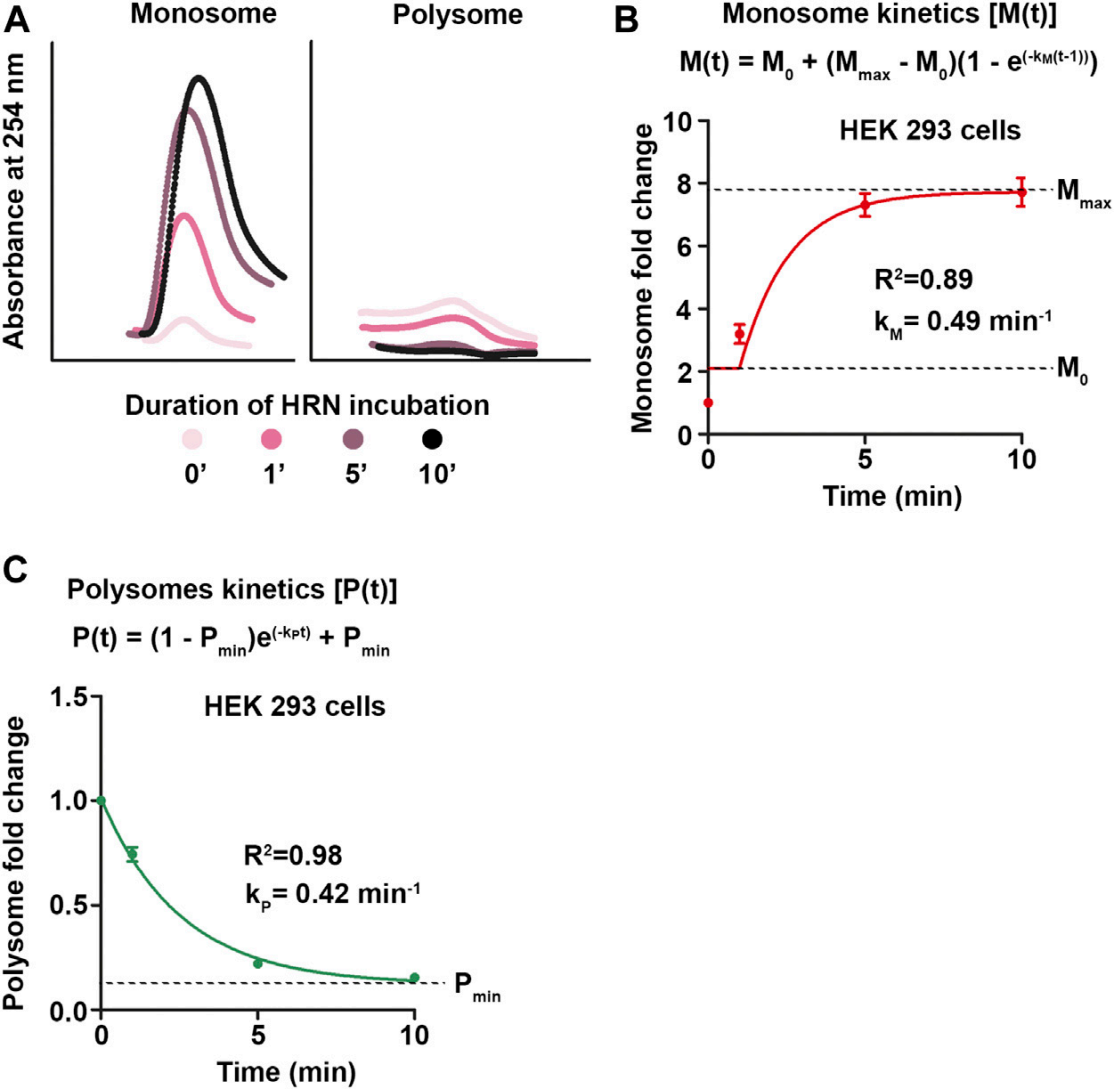
Monosome and polysome kinetics. **(A)** During 10 min HRN treatment, ribosomes accumulate as initiating ribosomes in the monosome fraction upon active elongation as polysomes. This causes changes in monosome and heavy polysome peaks during ribosome runoff. Light polysomes are not depicted. **(B,C)** Fold changes of monosomes **(B)** and polysomes **(C)** compared to CHX control profiles during ribosome runoff were calculated and plotted against the incubation time. These values were fitted with an exponential association and decay kinetics, respectively. Rates constants for monosomes (k_M_) and polysomes (k_P_) are depicted.

Next, we extracted monosome and polysome kinetics from our ribosome runoff experiments. HRN blocks the transition from initiating monosomes to elongating ribosomes. Hence, it does not inhibit initiating monosomes or elongating polysomes (Figure 3A). We used the increase in monosome intensity and the decrease in polysome levels to approximate their rate constants, respectively (Figures 3B, C). We plotted these values against the HRN incubation time and fitted the curves using an exponential association kinetics (Figure 3B) for monosomes and an exponential decay kinetics for polysomes (Figure 3C). Interestingly, we found that the rate constants of both ribosomal species are fairly similar (Figures 3B, C) showing the dependency between monosomes and polysomes (Chu et al., 2014). Together, our kinetics approach provide rate constants for global translation as well as for mono- and polysomes.

### 3.1 Short-term mTOR inhibition accelerates transition from monosomes to polysomes

The results from our kinetics approach prompted us to study dynamic changes in translational activity in cells under stress conditions. Therefore, we used different translational stressors such as the mTOR inhibitor Torin1, downregulation of the eukaryotic elongation factor 2 (eEF2) as well as starvation. We chose these stressors as they efficiently inhibit translation through different pathways. To investigate their impact on translation independent of possible changes in ribosome content, we used the same starting number of cells for control and treatment for all experiments. All downstream steps were perform volume-even.

**FIGURE 4:**
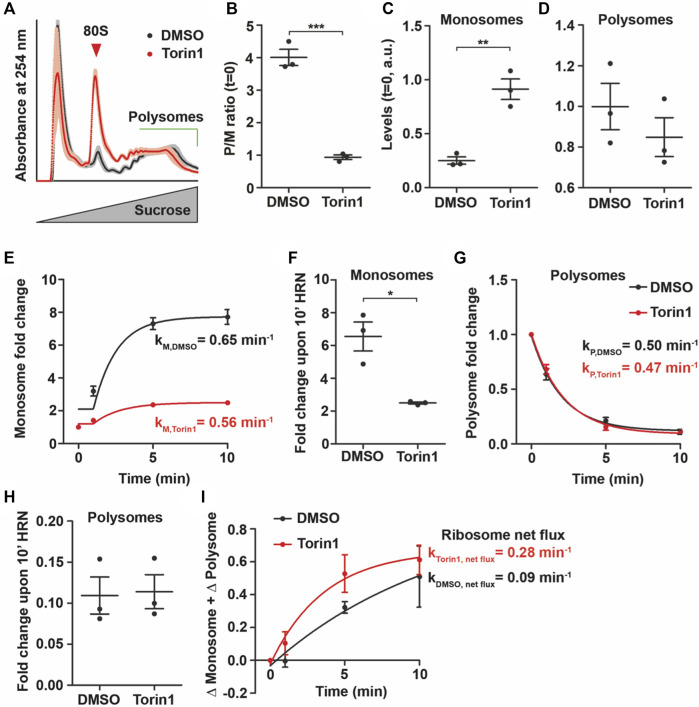
mTOR influences levels of monosomes. **(A)** Polysome profiles of DMSO and Torin1 treated cells after 30 min. Shadows represent SEM. **(B)** P/M(*t* = 0) ratio of DMSO and Torin1 treated cells. **(C,D)** Monosome **(C)** and polysome **(D)** levels of cells incubated with DMSO or Torin1 for 30 min **(E–H)** Monosome kinetics **(E)** and fold change **(F)** as well as polysome kinetics **(G)** and fold change **(H)** during ribosome runoff of DMSO and Torin1 treated cells. Rate constants for monosomes and polysomes are depicted. **(I)** Ribosome net flux kinetics for DMSO and Torin1 treated cells. Rate constants are depicted. *p*-values were calculated using Student’s *t*-test. *p < 0.05, **p < 0.01, ***p < 0.001, *n* = 3, dots represent biological replicates, data is represented as mean ± SEM.

First, we incubated HEK293T cells with different concentrations of Torin1 (Liu et al., 2010) for 30 min and tested for mTOR activity by immunoblotting against phospho(p)-mTOR and phosphorylation of its downstream target Rps6. As expected, we found that Torin1 decreased p-mTOR and phospho(p)-Rps6 levels (Supplementary Figures S4A, B) without significantly affecting total protein levels (Supplementary Figures S4C, D). To address the impact of Torin1 on translation dynamics, we used 100 nM Torin1 as this concentration led to significant changes in mTOR activity (Supplementary Figures S4A–D) but not in altered phosphorylation of eEF2 (Supplementary Figures S4E–H). We concluded therefore that 100 nM Torin1 would preferentially impact monosomes. As expected, we observed dramatic changes in the polysome profiles between cells treated with Torin1 and DMSO for 30 min (Figure 4A). We observed a significant decrease in the P/M ratio (*t* = 0) indicating reduced translational activity (Figure 4B). To further unravel the impact of mTOR inhibition on translation, we compared the amount of monosomes and polysomes between DMSO and Torin1 treatment. Surprisingly, we found that short-term incubation of Torin1 led to an increase in monosomes while polysomes showed similar levels between lysates from DMSO and Torin1 treated cells (Figures 4C, D). This finding raised the question whether total ribosome levels are also affected by short-term Torin1 treatment. To approximate for the number of ribosomes, we measure the area under the entire polysome profile including monosome, light and heavy polysome peaks. Interestingly, even though we used the same number of cells for DMSO and Torin1 treatment, mTOR inhibited HEK cells displayed a trend towards higher ribosome content (Supplementary Figure S4I). It has been shown that mTOR inhibition blocks translation of ribosomal proteins but leaves rDNA transcription unaffected leading to an imbalanced ribosomal protein and rRNA synthesis (Churchman et al., 2019). To unravel the impact of Torin1 on translation further, we performed translation kinetics with these cells. We analyzed the decay of translational activity over the time of HRN incubation and observed a strong reduction in the rate constant of translation speed k_P/M_ (Supplementary Figures S4J, K). In line with the role of mTOR in promoting translation (Laplante and Sabatini, 2012), we found that 100 nM Torin1 decreased the rate constant by ∼ 50% (Supplementary Figure S4K) leading to slower translational speed. As we observed that mainly monosomes were affected by Torin1 treatment, we speculated that also the monosome kinetics is more affected than the polysome kinetics. Indeed, we observed that the increase in monosomes is less pronounced in Torin1 treated cells compared to controls (Figures 4E, F). However, the rate constant was only slightly affected (Figure 4E). Surprisingly but in line with our quantification of polysomes (Figure 4D), we did not observe any difference in fold changes and rate constants of polysomes during ribosome runoff comparing DMSO and Torin1 treated cells (Figures 4G, H). To explain this conundrum and to understand how cells ensure consistent polysome speed even though levels of active monosomes decrease, we calculated the ribosome net flux. This kinetics takes into account monosome as well as polysome kinetics and represents the net flux of ribosomes from monosomes to polysomes or *vice versa*. For both conditions, we observed that the net flux is positive indicating a flux towards polysomes (Figure 4I). Interestingly, we found that this constant is more than 3 times higher for Torin1 treated cells than controls (Figure 4I). Importantly, this increase in ribosome net flux rate compensate the drop in monosome fold change which is 2.7 fold (Figure 4F) Together, these findings indicate that Torin1 treatment decreases the number of active monosomes but accelerates the net flux of the remaining monosomes towards polysomes in order to keep polysome kinectis constant.

### 3.2 Downregulation of eEF2 reduces levels of active monosomes

**FIGURE 5:**
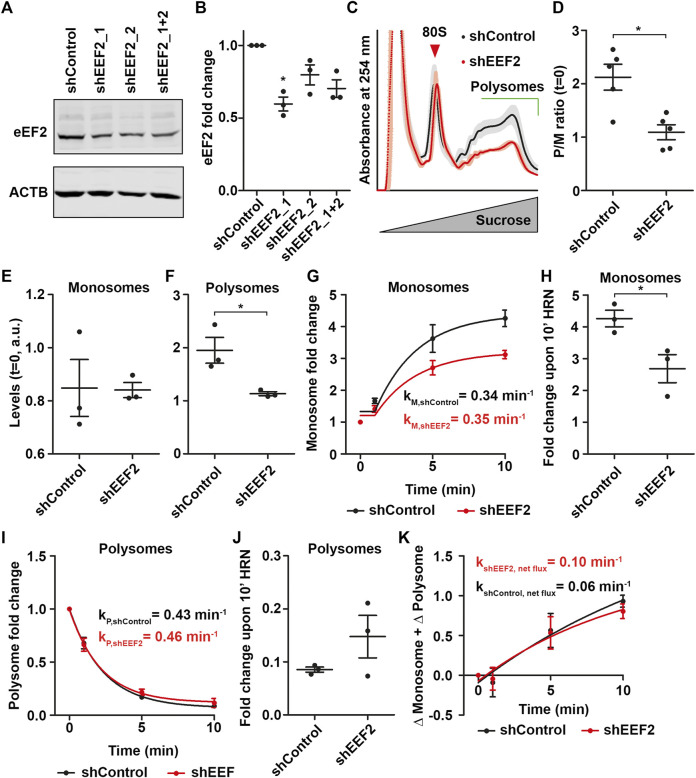
Monosomes compensate loss of eEF2. **(A,B)** Representative western blot against eEF2 using lysates from HEK cells transfected with shControl and different shEEF2 constructs **(A)** as well as quantification **(B)**. ACTB was used as loading control. **(C)** Polysome profiles of shControl and shEEF2 transfected cells. Shadows represent SEM. **(D)** P/M(*t* = 0) ratio of shControl and sheEF2 transfected cells. **(E,F)** Monosome **(E)** and polysome **(F)** levels of shControl and shEEF2 transfected cells. **(G–J)** Monosome kinetics **(G)** and fold change **(H)** as well as polysome kinetics **(I)** and fold change **(J)** during ribosome runoff of control and eEF2 depleted cells. Rate constants for monosomes and polysomes are depicted. **(K)** Ribosome net flux kinetics for shControl and shEEF2 transfected cells. Rate constants are depicted. *p*-values were calculated using one-sample *t*-test **(B)** or Student’s *t*-test **(D,F,H)**. *p < 0.05, *n* = 3, dots represent biological replicates, data is represented as mean ± SEM.

The impact of Torin1 on monosomes prompted us to investigate whether a similar effect can be observed for polysomes by depleting levels of an elongation factor. Therefore, we downregulated eEF2 using plasmids coding for shRNAs. We found a significant downregulation of eEF2 of ∼40% for shEEF2_1 (Figures 5A, B). For further experiments, we used shEEF2_1 and performed polysome profiling. As expected, we found that eEF2 knock-down also impairs steady-state P/M ratios (Figures 5C, D). In contrast to Torin1 treatment, we observed reduced polysome but not monosome levels in eEF2 knock-down (KD) lysates compared to controls (Figures 5E, F). Consequently, we also observed a drop in total ribosome levels as indicated by the reduced area under the polysome profile of eEF2 KD cells (Supplementary Figure S5A). This finding suggests that eEF2 depletion leads to a growth defect. Next, we performed translation kinetics and found translation speed was decreased (Supplementary Figures 5B, C). From these experiments we extracted monsome and polysome kinetics (Figures 5G, I) to test whether preferentially polysomes are affected in eEF2 depleted cells, analogous to monosomes in Torin1 treated cells. Surprisingly, monosomes showed lower fold changes but unaffected rate constants during runoff (Figures 5G, H), in eEF2 knock-down cells similar to Torin1 treated cells. Polysomes, in contrast, were unaffected in both fold changes and rate constants (Figures 5I, J). Moreover, we did not observe differences in ribosome net flux for shControl and shEEF2 transfected cells (Figure 5K).

Together, these findings point towards an adaption effect of the translational machinery. Stressors, that preferentially impair monosomes such as Torin1, cause an accelerated transition from monosomes to polysomes (Figure 4I) to stabilize polysomes. In contrast, stressors that reduce levels of polysomes drop the levels of active monosomes. These findings strongly suggest that cells adapt monosome levels and activity to stabilize polysomes under translational stress.

### 3.3 Amino acid depletion impacts monosome and polysome kinetics but allows for active elongation

**FIGURE 6:**
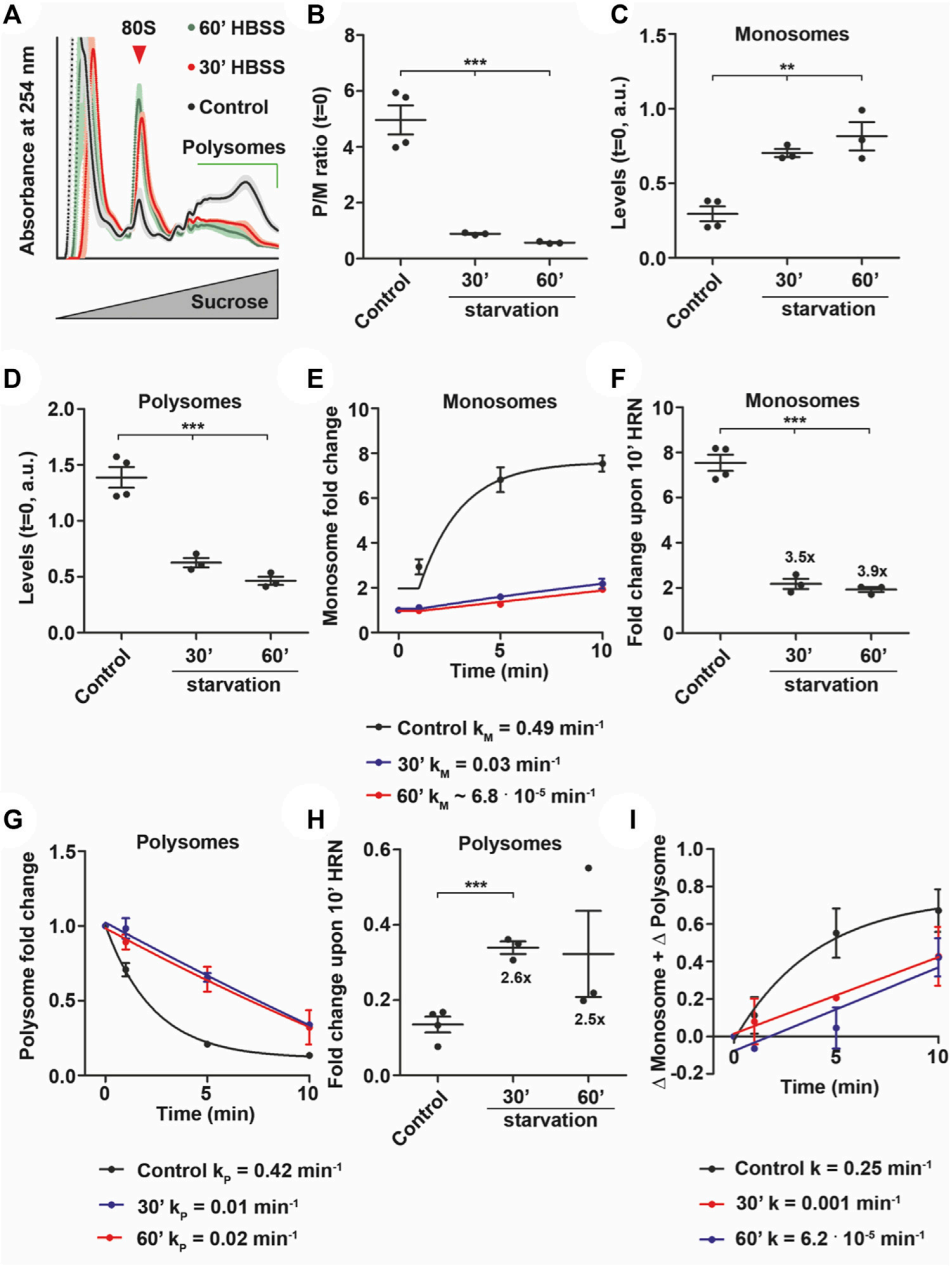
Amino acid depletion allows for translation elongation. **(A)** Polysome profiles of control and HBSS treated cells after 30 and 60 min. Shadows represent SEM. **(B)** P/M(*t* = 0) ratio of control and HBSS treated cells. **(C,D)** Monosome **(C)** and polysome **(D)** levels of control and HBSS treated cells. **(E–H)** Monosome kinetics **(E)** and fold change **(F)** as well as polysome kinetics **(G)** and fold change **(H)** during ribosome runoff of control and amino acid depleted cells. Numbers represent fold changes of mean compared to control cells. Rate constants for monosomes and polysomes are depicted. **(I)** Ribosome net flux kinetics for control and HBSS treated cells. Rate constants are depicted. *p*-values were calculated using One-way ANOVA with Tukey’s Multiple Comparison test **(B,C,D and F)** and Student’s *t*-test **(H)**. **p < 0.01, ***p < 0.001, *n* = 3, dots represent biological replicates, data is represented as mean ± SEM.

For the experiments described so far, we used stressors that preferentially target either monsomes (Torin1, Figure 4C) or elongating polysomes (eEF2, Figure 5F). To test how ribosomes respond to stressors that impair both monosome and polysomes, we depleted HEK cells from amino acids with Hank’s Balanced Salt Solution (HBSS) for 30, 60, 120, and 240 min and tested first for the activity of the nutrient-sensor mTOR (Laplante and Sabatini, 2012). As expected, p-mTOR decreased over the starvation time course (Supplementary Figures S6A, E). In contrast, we observed a significant upregulation of total mTOR protein upon 120 min (Supplementary Figures S6B, E). Moreover, we detected a strong increase in p-eEF2 levels (Supplementary Figures S6C, F) while total eEF2 levels were unaffected (Supplementary Figures S6D, F). These data suggest a global translational remodeling (Gambardella et al., 2020). Next, we performed translation kinetics focusing on the initial phase of starvation (30 and 60 min) to avoid side effects due to impaired cell viability. In line with our immunoblots, we observed a stark reduction in P/M ratios upon 30 and 60 min of starvation (Figures 6A, B). Importantly, Torin1 treated and starved cells exhibit a similar steady-state P/M ratio (compare Figure 4B with Figure 6B). However, we observed that lysates from starved cells displayed altered monosome and polysome levels (Figures 6C, D) in contrast to lysates from Torin1 treated cells where monosomes were preferentially affected (Figures 4C, D). Moreover, we did not detect a decrease in total ribosome levels by measuring the area under the entire polysome profile (Supplementary Figure S6G) which is supported by recent findings showing that ribosomes are protected from degradation during starvation (Shetty et al., 2023). In line with the altered monosome and polysome levels, we observed a 10 to 20 fold reduction in translation speed upon starvation for 30 and 60 min (Supplementary Figures S6H, I). As we detected lower activity of mTOR and increased phosphorylation of eEF2, we expected a complex impact on monosome and polysome kinetics. Indeed, we found that starvation drastically diminish the rate of monosome accumulation (Figure 6E). Interestingly, the effect was already observable upon 30 min of starvation (Figure 6E). This reduction led to a reduced number of active monosome as indicated by the drop in monosome fold change during runoff (Figure 6F). Similar to monosome rates, also polysome kinetics were found to be reduced (Figure 6G) which impacted also the fold change during runoff (Figure 6H). However, we noticed that the extent of changes was different. While the monosome fold change dropped almost 4 fold, polysome fold changes decreased by 2.6 fold (compare Figures 6F, H). Moreover, we still detected elongating polysomes upon 30 and 60 min of starvation, even though their rate was very low (Figure 6G). These data suggest that during starvation, cells reduce the activity of monosomes to favor polysomes. Supportive for this notion, upon 60 min of starvation, monosome rate constants and also ribosome net flux dropped further (Figures 6E, I) while polysome speed increased (Figure 6G). A finding that is supported by a previous study showing active translation even after 6 h (Gambardella et al., 2020).

### 3.4 Ribosome net flux kinetics predicts polysome levels in human cells

**FIGURE 7:**
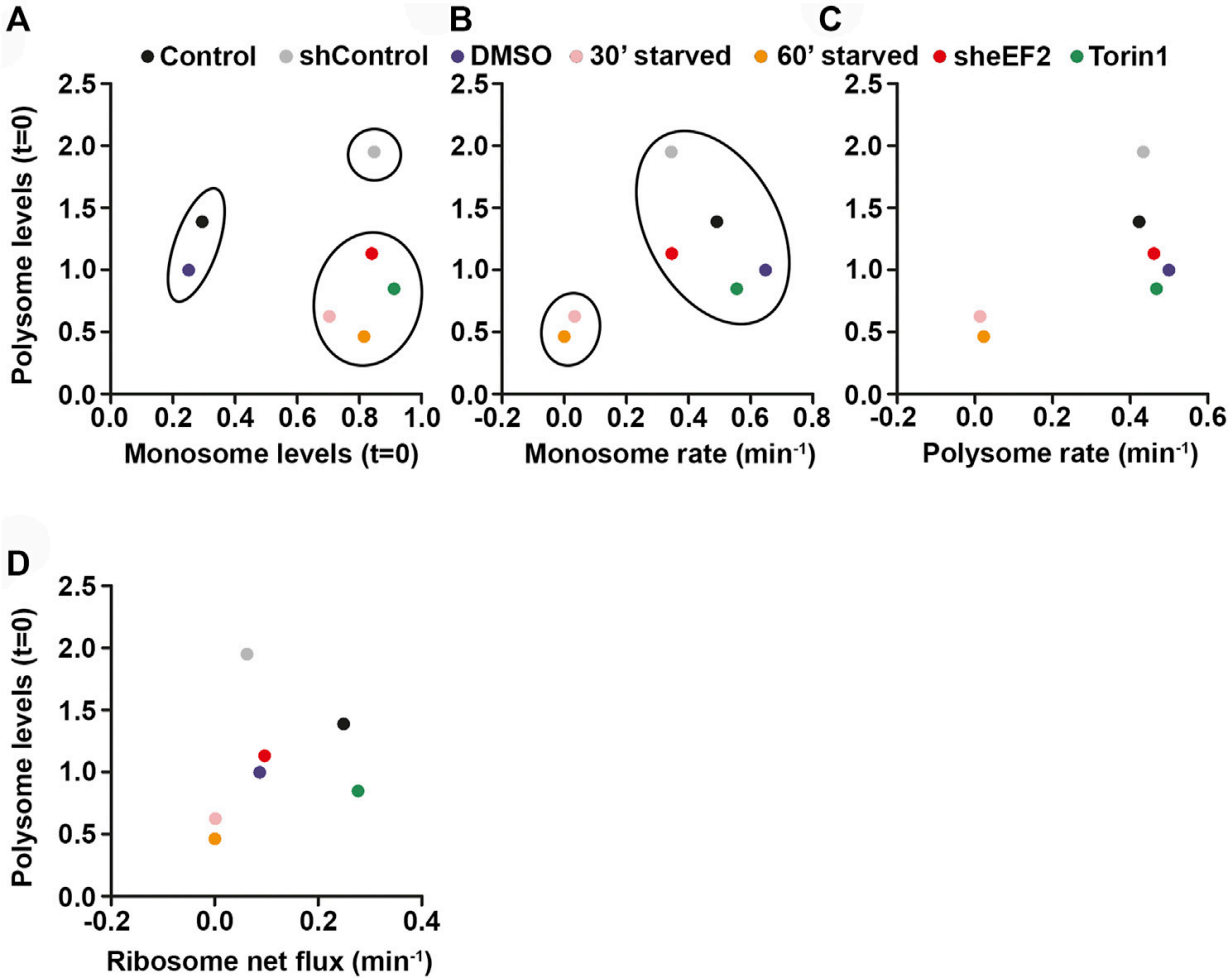
Monosomes buffer the costs of translational stress. **(A–D)** Correlations between monosome levels **(A)**, monosome rate constants **(B)**, polysome rate constants **(C)**, ribosome net flux constants **(D)** and polysome levels for different stress and control conditions. K-means clustering was used for **(A)** and **(B)**.

Having shown that cells favor polysomes over monsomes during translational stress, we wanted to use this data to proof which parameters predicts polysome levels and, in turn, translational activity in cells under stress conditions. Therefore, we correlated monosome levels (Figure 7A), monosome (Figure 7B) as well as polysome rate constants (Figure 7C) and ribosome net flux rate constants (Figure 7D) with polysome levels for the different translational stressors and control conditions we used. Moreover, we used k-means clustering to cluster the data points (Arthur and Vassilvitskii, 2007). Interestingly, we found that in the monosome-vs-polysome-level correlation (Figure 7A), data points cluster by stress rather than by the impact of the translational stressor on translation. In line with our findings, we found a more prominent shift along the monosome level axis than along the polysome level axis indicating that mainly monosome are affected during stress. Next, we tried to correlate monosome rate constants and polysome levels (Figure 7B). As we found that monosome rates were affected only in starved cells, we observed strong clustering of these cells in our plot (Figure 7B). Similarly, also the correlation between polysome rates and levels clustered stress and control conditions except for starved cells (Figure 7C) and does therefore not predict the polysome levels for the different conditions (Figure 7C). Based on our findings, we speculated that the ribosome net flux might be the best predictor of polysome levels as it includes monosome and polysome changes over time. Indeed, we found that the rate constant of ribosome net flux correlated best with polysome levels in HEK cells (Figure 7D). This finding suggests that the amount of ribosomes in polysomes is influenced by a balanced combination of monosome and polysome dynamics. In this scenario, faster monosome and slower polysome kinetics would increase polysomes and *vice versa*.

## 4 Discussion

### 4.1 Polysome profiling to study translation kinetics

Studying ribosome speed during translation is a prerequisite to understand co-translational folding trajectories and protein complex formation (Schieweck et al., 2016; Shiber et al., 2018). Here, we used a simple and straight-forward biochemical assay that utilizes ribosome runoff and polysome profiling to investigate mono- and polysome speed. We found that monosome accumulate with a rate of 0.49 min^−1^ during ribosome runoff which is similar to the initiation rate found by using the SunTag reporter constructs (Yan et al., 2016). In addition, based on our data, we calculated a polysome rate constant of 0.42 min^−1^ and a half-life time of 1.6 min. Ribosome profiling experiments have revealed an elongation rate of 3-4 codons/s for different cell types (Yan et al., 2016; Glock et al., 2021). Based on polysome sequencing results (Floor and Doudna, 2016), we calculated an average translation duration of 1.3–1.7 min. Hence, our translation kinetics experiments confirm published data on translation kinetics.

### 4.2 Quantification of mono- and polysomes during stress

Intense research in the last decades has shown that stress leads to translational shutdown of cells (Costa-Mattioli and Walter, 2020). These studies have mainly focused on the polysome-to-monosome ratios under certain stress conditions. However, whether stress affects certain ribosome assembly stages over others remained unaddressed; except for translationally inactive ribosomes (Brown et al., 2018; Shetty et al., 2023). Here, we set out to investigate monosome and polysome levels in lysates from cells that experienced different translational stress conditions. As we measured their abundance under volume-even conditions, we were able to determine levels of mono- and polysome independent of the total ribosome content that is regulated under certain stress conditions (Mayer and Grummt, 2006; Churchman et al., 2019). Importantly, while this approach allows for independent quantification of different ribosome complexes, normalization to the total ribosome content is useful to elucidate the relative contribution of monsomes and polysomes to translation.

### 4.3 Cells regulate mono- and polysome levels to balance translational stress

Translational stress mainly affects the eukaryotic initiation factor 2 α (eIF2α) as well as mTOR that both control translation activity (Ma and Blenis, 2009; Pakos-Zebrucka et al., 2016; Pernice et al., 2016). Importantly, translational shutdown is a dynamic process rather than a total block of protein synthesis. Different studies published so far have unraveled that cells allow for protein translation under stress condition which leads to upregulation of rescue and surveillance factors (Pakos-Zebrucka et al., 2016; Schneider et al., 2022). Moreover, to buffer the loss of translational activity, cells have evolved different mechanisms to stabilize their proteome and translatome (Schneider et al., 2020; Shetty et al., 2023). Therefore, they select mRNAs coding for stable, long-living proteins for translational attenuation (Schneider et al., 2020) or preserve ribosomes during stress (Shetty et al., 2023). These mechanisms are needed to allow for sufficient translation under stress conditions and for efficient cellular recovery upon stress release. Thus, the translational machinery itself is able to compensate translational inhibition. Supportive for this notion is our data on monosome and polysome levels and rates. The translational impairment induced by inhibiting mTOR or the downregulating eEF2 is buffered by monosomes to allow cells for active, nearly unaffected polysome rates. Therefore, monosomes show less activity in both stress paradigms. Even upon amino acid depletion, that showed a dramatic impact on monosomes and polysomes, monosomes were disproportionally more inactivated than polysomes. This finding is in line with the observation that endolysosomal and proteasomal proteins are newly translated during starvation to allow for cell survival (Gambardella et al., 2020). This effect is even more impressive as we detected increasing levels of phosphorylated eEF2 in starved cells. Another explanation for ongoing translation during starvation is the switch from polysome to monosome translated mRNAs (Schneider et al., 2022). In this scenario, elongating monosome might keep a certain level of translation activity but also reduce the energy costs for translation.

Together, these findings suggest that human cells balance the costs of reduced availability of crucial translation factors such as initiation or elongation factors as well as amino acids preferentially at the level of monosomes to allow for active ribosome elongation. This finding is in line with the general concept that ongoing translation elongation during stress is required to produce proteins needed for the cellular stress response (Spriggs et al., 2010). Undoubtedly, this process also requires a selection of mRNAs over others. This has been shown for different stressors (Thoreen et al., 2012; Pakos-Zebrucka et al., 2016; Gambardella et al., 2020). These mRNAs harbor certain features in their 5′- and 3′-untranslated regions (Spriggs et al., 2010) that allow them for efficient translation. Another plausible, not mutually exclusive possibility is that specialized ribosomes exist that activate translation of certain mRNAs under starvation. Specialized ribosomes have been mainly discussed in the context of development (Xue and Barna, 2012). It is known that certain ribosome interactors such as ribosomal proteins drive translation of certain mRNAs (Kondrashov et al., 2011). Thus, it is plausible that specialized ribosomes might be important to regulate the stress response. This possibility is particularly interesting as the ribosomal protein composition can be remodeled in response to stress (Fusco et al., 2021). In this context, ribosome levels represent another important regulatory feature. It is known that ribosome content is regulated by cells during brain development suggesting a regulatory role (Chau et al., 2018). Indeed, reducing ribosome content convey broad cellular changes (Khajuria et al., 2018; Cheng et al., 2019). Thereby, reduced ribosome levels affect certain subclasses of mRNAs over others (Khajuria et al., 2018; Cheng et al., 2019). We found that translation stressors can alter ribosome levels. Consequently, these altered ribosome levels might also contribute to translational remodeling and selection of mRNAs for translation. Future studies are clearly needed to unravel the molecular details of ribosome mediated control of translation during stress.

## Data Availability

The raw data supporting the conclusion of this article will be made available by the authors, without undue reservation.

## Supplementary material

Supplementary Figure 1 (Image 1) Polysome profiling allows for investigation of ribosome complexes. **(A/B)** HEK cells were treated with CHX or HRN for 10 min and lysates loaded on a 18%(w/v) to 50%(w/v) sucrose gradient. Representative immunoblots against Rpl7a and αTubulin **(A)** and quantifications **(B)** are depicted. Rpl7a served as ribosomal marker, αTubulin as cytosolic control protein Data were fit using Lorentzian distribution (for Rpl7a) or exponential decay (for αTubulin). n=3, data is represented as mean ± SEM.

Supplementary Figure 4 (Image 2 and 3) Torin1 inhibits mTOR. **(A–D)** Representative immunoblots against phosphorylated **(A)** as well as total **(C)** mTOR and Rps6 with respective quantifications **(B,D)**. ACTB was used as loading control. **(E–H)** Representative immunoblots against p-eEF2 **(E)** and eEF2 **(G)** and respective quantification **(F,H)**. ACTB was used as loading control. **(I)** Ribosome content of DMSO and 100 nM Torin1 treated cells. Ribosome levels were calculated from the area under the polysome profiles (80S + light polysomes + heavy polysomes). **(J)** Polysome profiles from DMSO and 100 nM Torin1 treated HEK cells upon 1, 5 and 10 min HRN treatment. Shadows represent SEM. **(K)** Kinetics of translational activity of DMSO and 100 nM Torin1 treated cells. Rate constants for the two curves are shown. *p*-values were calculated using one-sample t-test **(B,F)** or Student’s t-test **(I)**. **p* < 0.05, ***p* < 0.01, ****p* < 0.001, n=3, dots represent biological replicates, data is represented as mean ± SEM.

Supplementary Figure 5 (Image 4) Translation kinetics of shControl and shEEF2 transfected cells. **(A)** Ribosome content of shControl and shEEF2 transfected cells. Ribosome levels were calculated from the area under the polysome profiles (80S + light polysomes + heavy polysomes). **(B)** Polysome profiles from shControl and shEEF2 transfected HEK cells upon 1, 5 and 10 min HRN treatment. Shadows represent SEM. **(C)** P/M ratio were fitted using an exponential decay kinetics. *p*-values were calculated using paired Student’s t-test. **p* < 0.05, n=3–5, dots represent biological replicates, data is represented as mean ± SEM or as data pairs.

Supplementary Figure 6 (Image 5) Amino acid starvation induces phosphorylation of eEF2. **(A–D)** Representative immunoblots against p-mTOR **(A)**, mTOR **(B)**, p-eEF2 **(C)** and eEF2 **(D)** for different starvation durations. ACTB was used as loading control. **(E,F)** Quantification of immunoblots shown in **(A–D)** for p-mTOR and mTOR **(E)** as well as p-eEF2 and eEF2 **(F)** for different starvation durations. **(G)** Ribosome content of control and starved HEK cells. Ribosome levels were calculated from the area under the polysome profiles (80S + light polysomes + heavy polysomes). **(H)** Translation kinetics of control and 30 min as well as 60 min starved HEK cells. Rate constants are depicted. **(I)** Polysome profiles from control and starved cells upon 1, 5 and 10 min HRN treatment. Shadows represent SEM. *p*-values were calculated using one-sample t-test. **p* < 0.05, ***p* < 0.01, n=3–4, dots represent biological replicates, data is represented as mean ± SEM.
