# Supplementary figures and images for: Monosomes buffer translational stress to allow for active ribosome elongation
Source: Front Mol Biosci. 2023 May 26;10:1158043. doi: 10.3389/fmolb.2023.1158043 (PMC10253174; doi:10.3389/fmolb.2023.1158043)

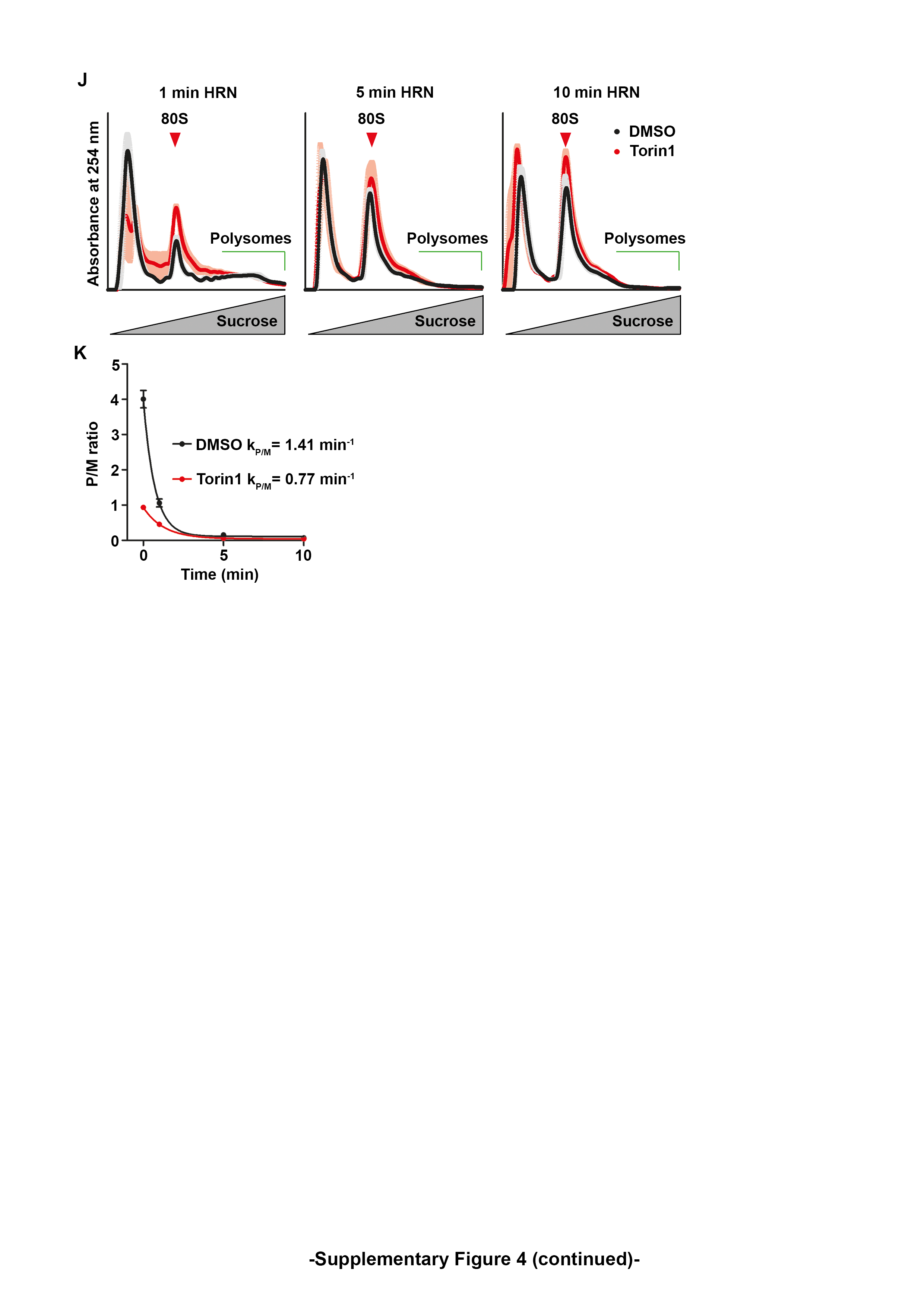

Supplement: Supplementary file 1 [file Image3.tif]

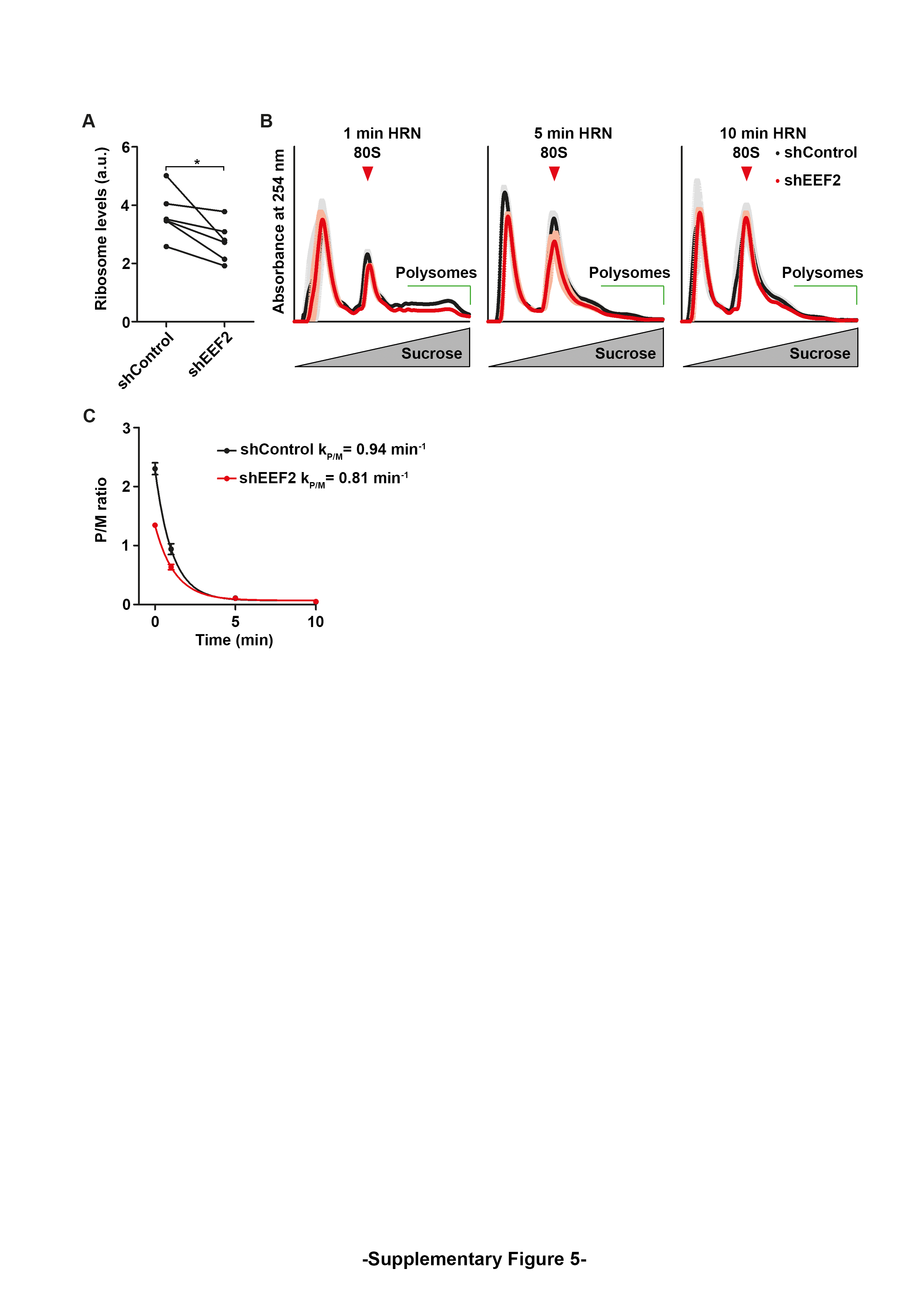

Supplement: Supplementary file 2 [file Image4.tif]

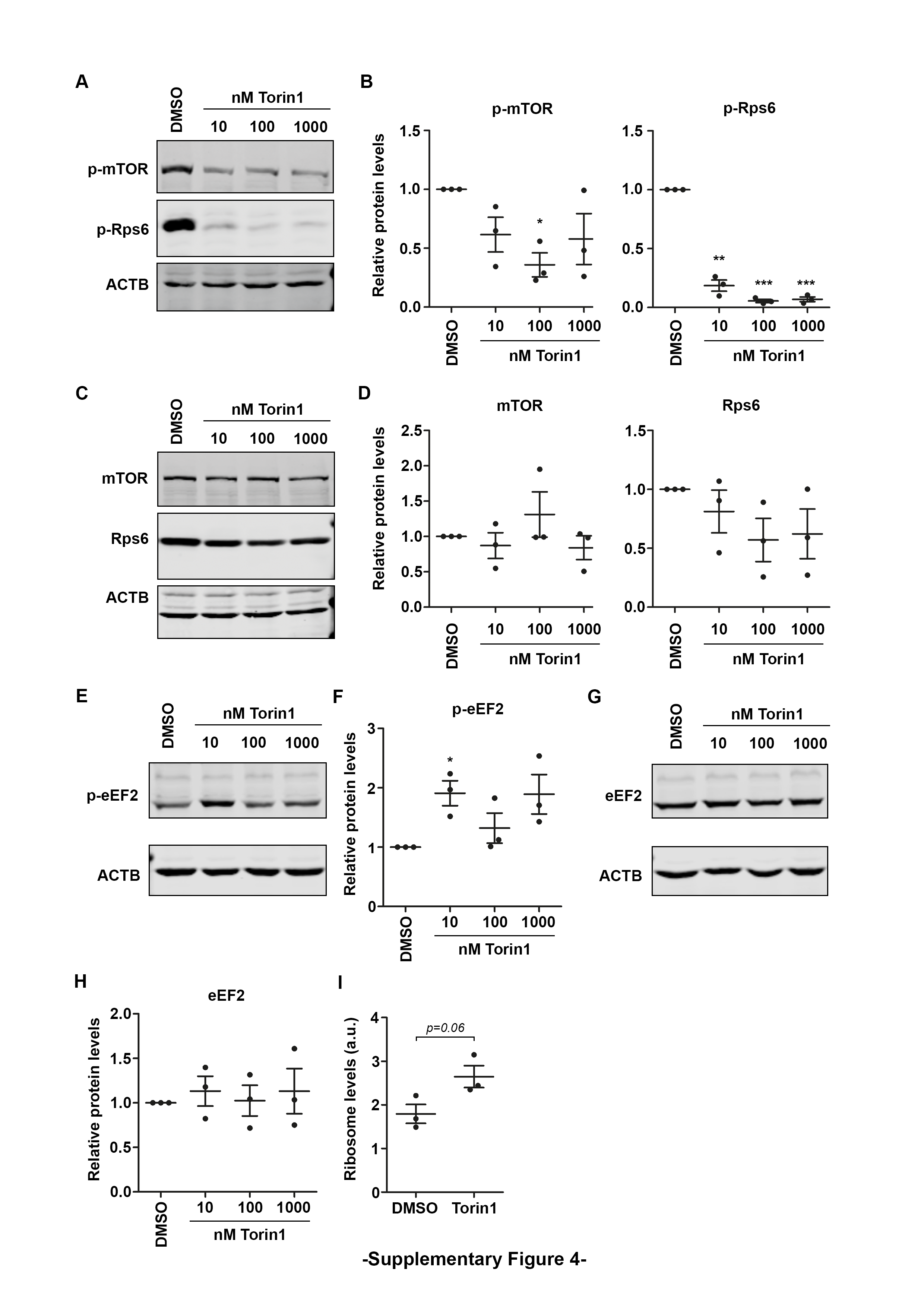

Supplement: Supplementary file 3 [file Image2.TIF]

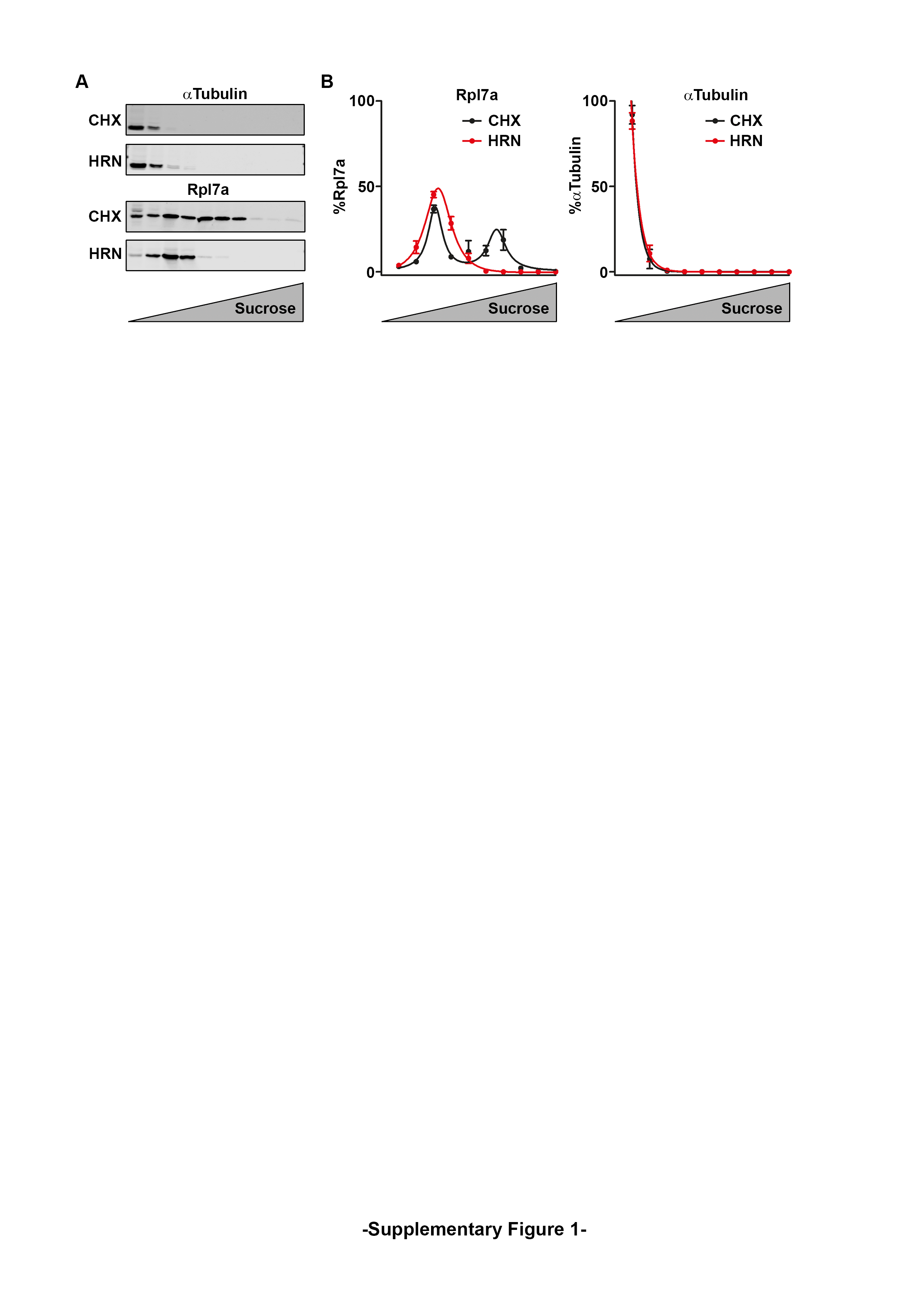

Supplement: Supplementary file 4 [file Image1.tif]

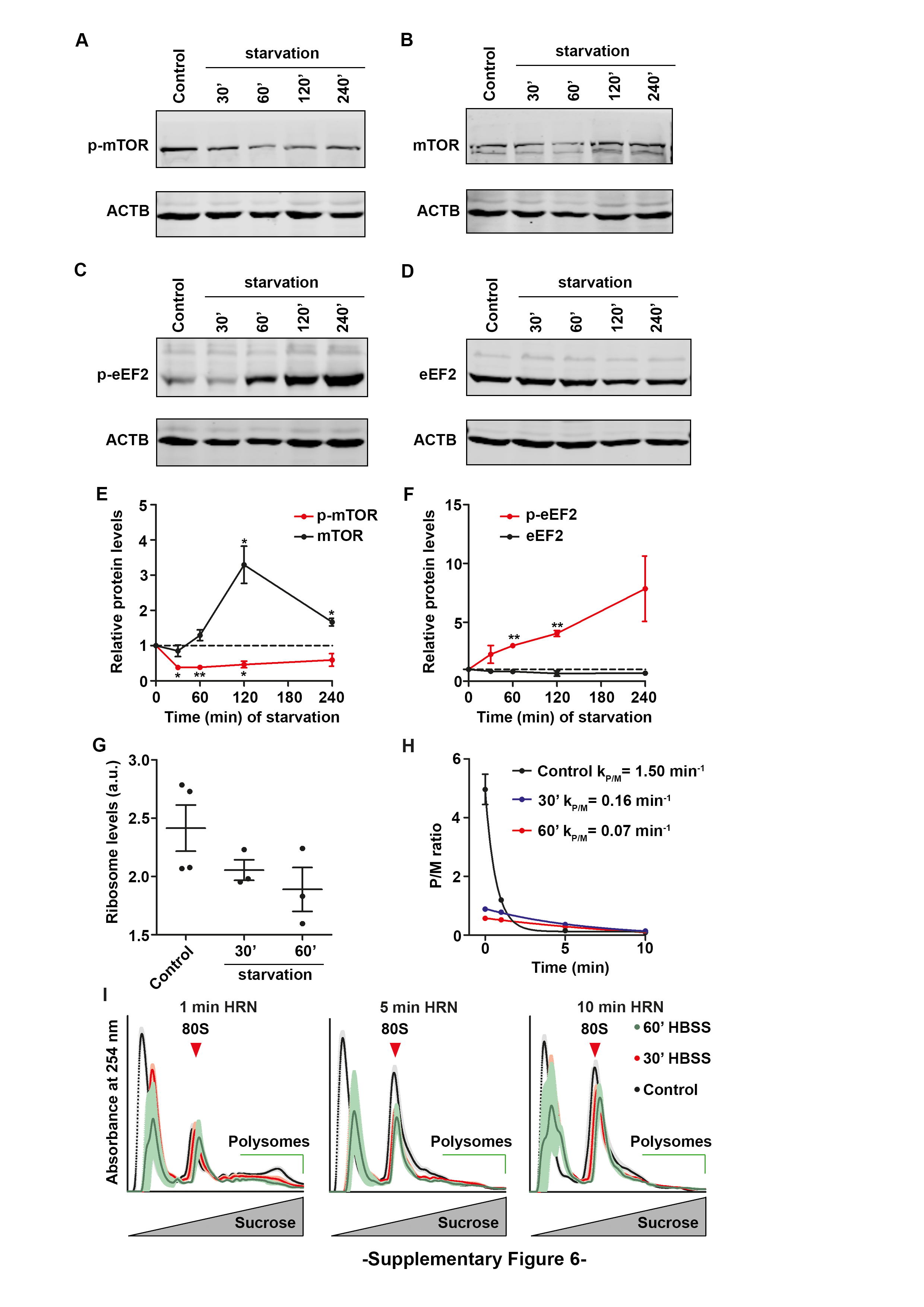

Supplement: Supplementary file 5 [file Image5.tif]
